# Risk of Endometrial Cancer in Women With Pelvic Inflammatory Disease: A Nationwide Population-Based Retrospective Cohort Study

**DOI:** 10.1097/MD.0000000000001278

**Published:** 2015-08-28

**Authors:** Teng-Kai Yang, Chi-Jung Chung, Shiu-Dong Chung, Chih-Hsin Muo, Chao-Hsiang Chang, Chao-Yuan Huang

**Affiliations:** From the Surgery Department (T-KY), Yonghe Cardinal Hospital; School of Medicine, College of Medicine (T-KY, S-DC), Fu Jen Catholic University, New Taipei City; Department of Health Risk Management (C-JC), College of Public Health, China Medical University; Department of Medical Research (C-JC), China Medical University Hospital, Taichung; Division of Urology Department of Surgery (S-DC), Far Eastern Memorial Hospital, New Taipei; College of Medicine (C-HM, C-HC), China Medical University; Management Office for Health Data (C-HM), China Medical University and Hospital; Department of Urology (C-HC), China Medical University and Hospital, Taichung; and Department of Urology (C-YH), National Taiwan University Hospital, Taipei, Taiwan.

## Abstract

To investigate the association between pelvic inflammatory disease (PID) and endometrial cancer (EC).

We conducted a nationwide population-based retrospective cohort study, and data were obtained from the National Health Insurance Research Database. We defined 41,065 patients with PID as the PID cohort and 82,130 randomly selected patients as the control cohort through frequency matching by age and index year. PID and EC were diagnosed in accordance with the International Classification of Diseases, Ninth Revision, and Clinical Modification. Cox proportional hazards regression and Kaplan-Meier method were used in the analysis.

Incidence rates of 16.1 and 9.6 per 100,000 person-years and mean follow-up durations of 4.84 and 6.63 years were observed in the PID and non-PID cohorts, respectively. After adjusting for potential risk factors, the PID cohort had a 1.79-fold higher risk of developing EC than the non-PID cohort. The incidence of EC increased with age, particularly for those aged >50 years (HR = 2.45, 95% CI = 1.29-4.65). Higher EC risk was also observed in the PID cohort with hypertension than in the non-PID cohort.

The results of this large-scale population-based study showed an increased risk of EC in PID patients, particularly in older patients or those with hypertension. Future large-scale clinical trials are warranted to clarify the function of medication in PID-related EC progression.

## INTRODUCTION

Endometrial cancer (EC), the leading gynecologic malignancy in developed countries, is predisposed to exogenous estrogen use and metabolic risks such as obesity, hypertension, and diabetes.^1-3^ In Taiwan, the incidence and mortality rate of EC have been rising in the past years, with age-adjusted incidence rates of 11.25 and 189 per 100,000 women in 2010 (Bureau of Health Promotion, 2010).

Pelvic inflammatory disease (PID) is a common gynecologic disease in which infections, such as endometriosis, salpingitis, pelvic peritonitis, and abscess, arise from the lower genital tract to the upper sites and may cause chronic pain and infertility.^4^ PID usually affects patients of low socioeconomic status and with comorbidities, such as cardiovascular disease, endometriosis, diabetes, chronic liver disease, and rheumatic disease.^5,6^

Inflammation, which is driven by various mediators, has been associated with cancer development.^7^ The association between PID and gynecologic or nongynecologic malignancies, including cervical cancer, ovarian cancer, and colorectal cancer, has been investigated.^6,8,9^ Lin et al^6^ found a high ovarian cancer risk in PID patients, especially in those with 5 plus episodes. PID as a risk factor for EC has been sporadically reported,^10^ but evidence of the link between PID and EC remains scarce. Thus, we conducted a large-scale and nationwide study to investigate the role of PID in EC development.

## METHODS

### Data Source

The Taiwan National Health Insurance Research Database (NHIRD) was a longitudinal database set up by the Bureau of National Health Insurance on March 1, 1995. Under the National Health Insurance program, 99% of the island's population receives all forms of health care services, including ambulatory care, outpatient and inpatient treatments, dental services, and physician-provided services. This database contained outpatient and inpatient claims from 1996 to 2010. We obtained the Longitudinal Health Insurance Database (LHID), which is a subdatabase that includes 1 million randomly selected insurants in 2000 registry of beneficiaries. We used LHID and the catastrophic illness patient registry (CIPR) for this study and linked them through insurant identification. The inclusion of patients in CIPR was based on histological report or typical radiological features. Insurant identification was encrypted before being sent to the researcher.

### Study Participants

We collected the data of 41,298 women newly diagnosed with PID in accordance with the International Classification of Diseases, Ninth Revision, Clinical Modification (ICD-9-CM): 614 in 1998 to 2010. PID women with malignant history (ICD-9-CM: 140-208 from CIPR) or developed metastasis (ICD-9-CM: 196-199 from CIPR) were excluded. All 41,065 PID women were selected as the PID cohort, and the date for PID was defined as the index date. Non-PID controls were selected from women without PID and malignant history before the index date. Non-PID controls were mostly selected from women who visited obstetric or gynecological clinics but without PID and malignancy by the index date after routine checkups. The control cohort was frequency matched with age stratum (every 5 years) as the PID cohort at a 2:1 ratio.

### Outcome and Covariate

The 2 cohorts were followed from the index date to the occurrence of EC (ICD-9-CM: 182). Cohort members who did not develop EC were followed to the date of withdrawal from this program or the end of 2010. Covariates included monthly age, monthly income, occupation, urbanization of living condition, and comorbidities, such as coronary artery disease (ICD-9-CM: 410-414), endometriosis (ICD-9-CM: 617), diabetes (ICD-9-CM: 250), hypertension (ICD-9-CM: 401-405), hyperlipidemia (ICD-9-CM: 272), cirrhosis (ICD-9-CM: 571), and rheumatic disease (ICD-9-CM: 714 from CIPR). All comorbidities were defined before the index date and confirmed with at least 3 medical visits to increase the validity of the diagnoses. Urbanization level was adopted from the study by Chang et al.^11^

### Statistical Analysis

All statistical analyses were performed using SAS statistical software (version 9.3 for Windows; SAS Institute, Inc., Cary, NC). Chi-square test was used to calculate the differences in baseline characteristics between the PID and non-PID cohorts. The incidences for EC (per 10,000 person-years) between the 2 cohorts were calculated. The hazard ratio and 95% confidence interval (CI) for EC were assessed by comparing the 2 cohorts through Cox proportional hazards regression. The multivariable model included controlling age, monthly income, occupation, hyperlipidemia, hypertension, diabetes, and coronary artery disease. The stratified analysis was estimated in accordance with age group (≤40, 41-50, and >50 years), follow-up duration (<2, 2-4, and ≥5 years), or comorbidity. Kaplan-Meier analysis was used to plot the cumulative incidence, and log-rank test was used to test the difference between the 2 cohorts. Statistical significance was considered at *P* < 0.05 (2-tailed).

### Ethics

To protect personal information, this study was approved by the Institutional Review Board of China Medical University Hospital (CMU-REC-101-012).

## RESULTS

### Comparison of Demographic Profiles Between PID and Non-PID Cohorts

We collected the data of 123,195 study participants, including 41,065 for the PID cohort and 82,130 for the non-PID cohort. The majority of the PID women were aged < 40 (73.4% vs 26.6%) years, and the mean age was 33.4 years (standard deviation, SD = 11.8). Compared with the non-PID cohort, more PID women were with middle income (81.7% vs 63.6%), blue collar (20.9% vs 17.7%), living in lower urbanized areas (12.1% vs 10.5%), and had comorbidity, except for rheumatic disease (0.06% vs 0.09%) (Table 1).

**TABLE 1:**
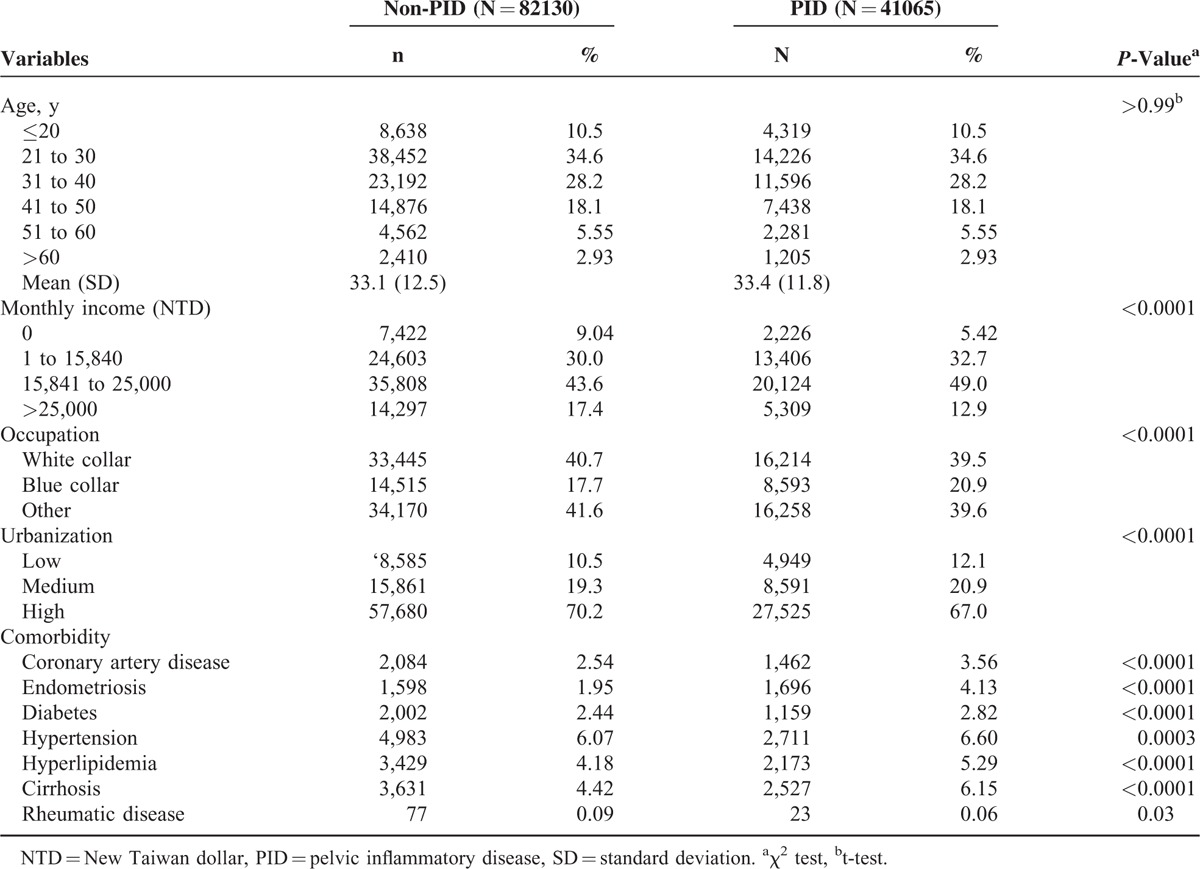
Comparison of Baseline Characteristics Between PID and Control Groups

### Association Between Plasma PID and EC Risk

**FIGURE 1:**
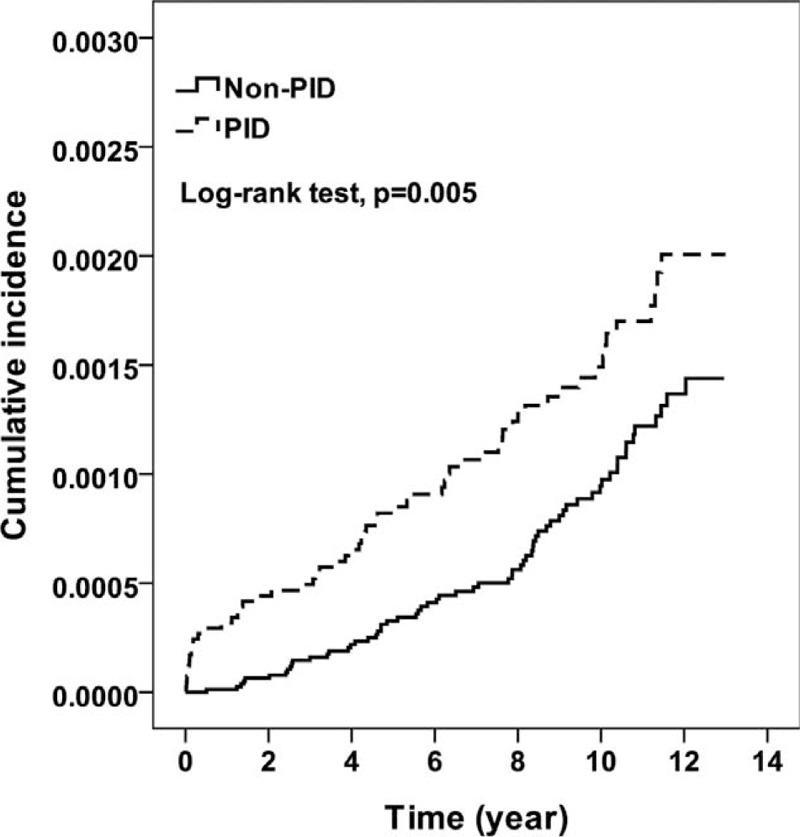
Kaplan-Meier curve for the incidence of EC. EC = endometrial cancer, PID = pelvic inflammatory disease.

During the 10-year follow-up period, 59 and 64 incidence cases of EC were reported in the PID and non-PID cohorts, respectively. The corresponding mean follow-up times to incidence were 4.84 (SD, 3.72) and 6.63 (SD, 3.19). The Kaplan-Meier survival analysis showed that the PID cohort had significantly higher incidence of EC than the non-PID cohort (log-rank *P* = 0.005, Figure 1). The rate of PID women who developed EC was higher (16.1 vs 9.6 per 100,000 person-years) than that of non-PID women, and the risk of PID women was 1.79-fold greater than that of non-PID women, as shown in the multivariable Cox proportional hazards regression model (Table 2). For age-specific incidence and risk, the incidence increased with age in both cohorts, particularly with the significant risk at age >50 years (HR = 2.45, 95% CI = 1.29-4.65). In the analysis of the follow-up duration strata, PID women had a higher risk than non-PID women, but only patients who were followed for <2 years were significantly different (HR = 7.91, 95% CI = 2.92-21.4). PID women with hypertension had a 3.06-fold higher risk than non-PID women with hypertension after adjusting for other potential factors (95% CI = 1.34 to −6.95; *P* < 0.01).

**TABLE 2:**
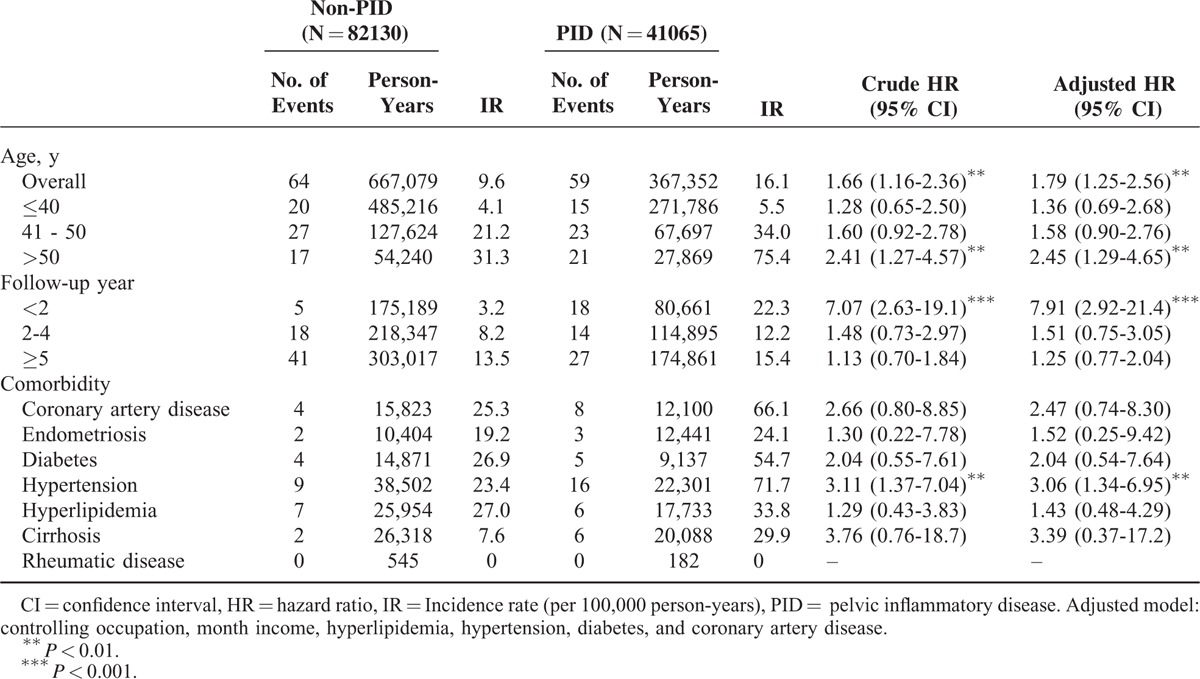
Incidence and Hazard Ratio of Endometrial Cancer

Furthermore, we evaluated the effect of PID and hypertension on the risk of EC (Table 3). The PID cohort with hypertension had significantly higher adjusted HR of EC than the non-PID cohort without hypertension (adjusted HR = 2.36, 95% CI = 1.22 to −4.57). However, PID and hypertension showed no significant influence on the risk of EC (*P* = 0.1).

**TABLE 3:**
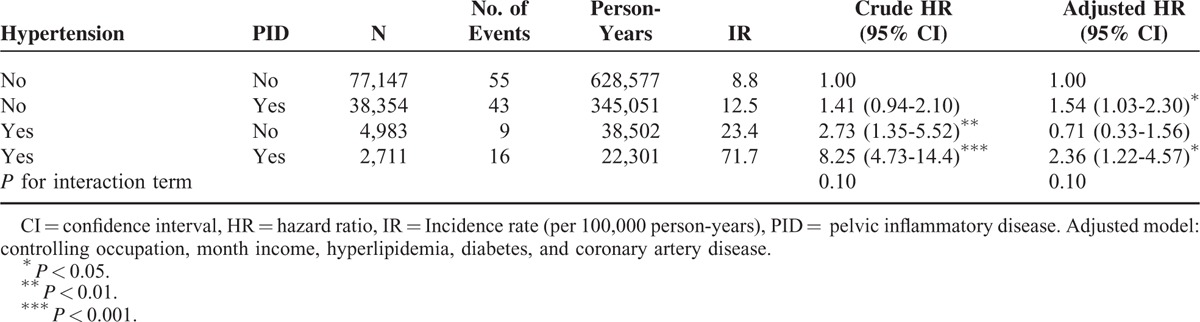
Incidence and Hazard Ratio of Endometrial Cancer

## DISCUSSION

To the best of our knowledge, this study is the first large-scale and nationwide one to confirm PID as an independent risk factor for EC in Taiwan. We concluded that the PID cohort, especially PID patients aged >50 years or with hypertension history, had higher EC risk after controlling potential confounders (overall hazard ratio 1.79) than the non-PID cohort.

In the present study, PID patients aged >50 years had 2.45-fold higher risk of EC than non-PID patients. Lin et al^6^ reported that PID patients aged ≤35 years are at higher risk of ovarian cancer than PID patients aged >35 years because of the higher rates of sexual activity in the former cohort. Different from the peak age of ovarian cancer incidence , that of EC in the present study was 50 to 60 years of age. This result is similar to the previous statistical data.^12^ A high risk for EC was also found in old patients with short follow-up durations; this result indicates that carcinogenesis relies on the immune susceptibility of individuals instead of the frequency of sexual exposure.^13^ Patients with hypertension and other metabolic factors, such as central obesity and diabetes, reportedly have a high risk for EC. A large case-control study showed that hypertension exerts the strongest effect on EC risk (odds ratio, 6.3) among metabolic factors.^14^ In the present study, hypertension history had the strongest effect modifier of the PID effect for EC risk among all comorbidities. In addition, hypertension history is the only factor involved in the effect modification of PID risk on EC among all metabolic abnormalities. Recent clinical studies have demonstrated that essential hypertension positively correlates with biomarkers of oxidative stress, a condition that promotes endothelial dysfunction and inflammation.^15^ Hypertension reportedly consolidates PID inflammation and magnifies EC risk through the oxidative stress pathway instead of through insulin resistance.

Inflammation is a key step in the endometrium remodeling cycle, in which cytokines are involved in the change of endometrial mucosa.^16^ Inflammatory cells may promote cell proliferation, inhibit apoptosis, and contribute to genetic alteration.^17^ Meanwhile, endometrial cells chronically affect immune-related cytokines and growth factors.^18^ Studies also correlated high EC risk to elevated levels of proinflammatory markers, especially in the COX pathway.^19,20^ Certain genetic polymorphisms in genes observed in the inflammatory pathway increase the genetic susceptibility to EC,^21^ which may be triggered through chronic inflammation via bacterial- or viral-like PID.^22^

This dualistic model of endometrial carcinogenesis is well known and is based on a hypothesis by Bokhman.^23^ Most ECs are sporadic; thus, environmental predisposing factors such as exogenous estrogen and chronic inflammation but not genetic alteration may influence endometrial carcinogenesis. Obesity, a state of low-grade chronic inflammation with elevated adiposity-related inflammatory cytokines such as TNF-α, IL-6, and C-reactive protein, may contribute to endometrial carcinogenesis and confound the results in the present study.^20,24^ Although no data on weight or waist circumference were included because of the NHIRD limitation, we applied comorbidities such as hyperlipidemia, hypertension, and diabetes as metabolic surrogate markers in the adjusted regression models.

Anti-inflammatory medication is well known as a potential cancer chemoprevention agent. NSAIDs such as celecoxib, sulindac sulfide, and acetaminophen combined with progestin inhibit cell growth induce apoptosis in the ovarian epithelium, and lower cancer risk.^25^ A few studies suggested that aspirin is associated with reduced EC risk, especially among obese, nonsmoking, and estrogen-mediated women.^19,26^ Aside from antimicrobial therapy, regular, long-term aspirin may also be beneficial for patients with PID and comorbidities such as cardiovascular disease, endometriosis, diabetes, chronic liver disease, and rheumatic disease. However, rigorous study is required to clarify the results.

The present study has several strengths. First, data were collected from a cohort of the NHIRD, which covered more than 98% of the total Taiwan population, thereby minimizing the possibility of recall bias or biased follow-up. Second, the diagnosis of EC was confirmed by pathology reports, and the results of EC were extracted from Taiwanese National Cancer Registry. Although the selection of cases was not randomized, the large-scale study population chosen by the International Classification of Diseases code prospectively lowered the selection bias. Finally, the stratified data enabled us to visualize the distribution of EC by the effects between PID and potential confounders. The latent period for EC, which was defined as the time between the first PID episode and diagnosis of EC, was around 5 years in our non-PID cohort. However, the latent period was surprisingly shortened to approximately 2 years in our PID participants, which emphasizes the strong effect of PID on EC risk.

However, this study has also some limitations. NHIRD did not contain detailed information on the patient's reproductive factors, such as age at menarche, age at menopause, parity, time since last full-term pregnancy, and use of oral contraceptives.^27^ Therefore, the results of the present study were not adjusted to consider these factors, which may confound the association between PID and EC. Furthermore, several time-variant covariates were analyzed at single measurement. Therefore, we assumed these variables as constant over the study period. In addition, the study population was Asian. Hence, some sexuality factors that can affect PID notably differed from non-Asian ethnicities; these factors include age of sexual debut, frequency of sexual intercourse, or oral contraceptive usage.^28^ Moreover, the results of the present study might not allow precise extrapolation. Another limitation was the lack of cancer staging, which could be a prognostic factor for EC. Patients of advanced stage at diagnosis imply the aggressive behavior of EC, which needs attention and proper management. However, the present results confirmed that PID was a strong, independent risk factor for endometrial carcinogenesis. Therefore, clinicians must be aware of the association and seek for optimal treatments, in which chemoprevention with anti-inflammatory agents may play a role in the near future.

In summary, this large-scale and population-based study indicated the increased risk for EC of PID patients, particularly old patients or those with hypertension. Future large-scale clinical trials are needed to clarify the role of medication in PID-related EC progression.
